# Optimizing the bioenergy water footprint by selecting SRC willow canopy phenotypes: regional scenario simulations

**DOI:** 10.1093/aob/mcz006

**Published:** 2019-02-13

**Authors:** Benjamin Richard, Goetz M Richter, Marianna Cerasuolo, Ian Shield

**Affiliations:** 1 Department of Sustainable Agriculture Sciences, Rothamsted Research, Harpenden, UK; 2 Department of Biological and Environmental Sciences, School of Life and Medical Sciences, University of Hertfordshire, Hatfield, UK; 3 Department of Mathematics, University of Portsmouth, Lion Terrace, Portsmouth, UK

**Keywords:** Bioenergy, canopy type, climate change, evapotranspiration, marginal soils, water use efficiency, woody biomass

## Abstract

**Background and Aims:**

Bioenergy is central for the future energy mix to mitigate climate change impacts; however, its intricate link with the water cycle calls for an evaluation of the carbon–water nexus in biomass production. The great challenge is to optimize trade-offs between carbon harvest and water use by choosing cultivars that combine low water use with high productivity.

**Methods:**

Regional scenarios were simulated over a range of willow genotype × environment interactions for the major UK soil × climate variations with the process-based model LUCASS. Soil available water capacity (SAWC) ranged from 51 to 251 mm and weather represented the north-west (wet, cool), north-east (dry, cool), south-west (wet, warm) and south-east (dry, warm) of the UK. Scenario simulations were evaluated for small/open narrow-leaf (NL) versus large/closed broad-leaf (BL) willow canopy phenotypes using baseline (1965–89) and warmer recent (1990–2014) weather data.

**Key Results:**

The low productivity under baseline climate in the north could be compensated by choosing BL cultivars (e.g. ‘Endurance’). Recent warmer climate increased average productivity by 0.5–2.5 t ha^−1^, especially in the north. The modern NL cultivar ‘Resolution’ had the smallest and most efficient water use. On marginal soils (SAWC <100 mm), yields remained below an economic threshold of 9 t ha^−1^ more frequently under baseline than recent climate. In the drought-prone south-east, ‘Endurance’ yielded less than ‘Resolution’, which consumed on average 17 mm year^−1^ less water. Assuming a planting area of 10 000 ha, in droughty years between 1.3 and 4.5 × 10^6^ m^3^ of water could be saved, with a small yield penalty, for ‘Resolution’.

**Conclusions:**

With an increase in air temperature and occasional water scarcities expected with climate change, high-yielding NL cultivars should be the preferred choice for sustainable use of marginal lands and reduced competition with agricultural food crops.

## INTRODUCTION

Biomass is a central part of the renewable energy mix for fuel, heat and power, and has attracted continuing R&D investment into the development of advanced, lignocellulosic energy crops (DOE, 2014; Karp *et al.*, 2014). Willows (*Salix* spp.), grown in short-rotation coppice (SRC), are an important source of biomass, bring multiple environmental benefits and offer a potential additional source of income for farmers (Busch, 2017). However, due to competing land demands (Li *et al.*, 2013) and concerns about food security (Karp and Richter, 2011), perennial energy crops should be allocated to agricultural land that is non-profitable for food, to increase profitability and sustainability of the whole farm (Nair *et al.*, 2017). When grown on marginal land they are expected to achieve maximum climate-change mitigation (Robertson *et al.*, 2017), e.g. carbon sequestration (Agostini *et al.*, 2015). However, there are concerns over potential negative impacts. Although it has been demonstrated that willows bring positive benefits to water quality (Wu *et al.*, 2012), their impacts on groundwater recharge still need to be addressed quantitatively (DOE, 2014). In fact, it has been suggested that to contain the potential increase in fresh water usage in large-scale bioenergy production (Gheewala *et al.*, 2011), there is a need for explicit water-protection policies (Bonsch *et al.*, 2016) that are based on quantitative evidence about productivity, water use and water-productivity trade-offs (Silalertruksa and Gheewala, 2018).

Short-rotation coppice plantations offer an increasing mitigation potential to reduce atmospheric CO_2_ accumulation (Amichev *et al.*, 2012; Hammar *et al.*, 2014). In North America, yields of new cultivars have increased from 12 to 17.4 t ha^−1^ (Amichev *et al*., 2015), likely out-performing native willow species (Zamora *et al.*, 2014). However, annual yields on marginal soils in Europe are inevitably smaller (Guénon *et al.*, 2016), often due to limited water availability (Larsen *et al.*, 2016). Overall, annual on-farm yields can range from 2 to 15 t ha^−1^ (Searle and Malins, 2014) with average dry matter (DM) yields of <8 t ha^−1^ (Stolarski *et al.*, 2011, 2014). For the future, investigations need to focus on genotype × environment (G × E) (× management) interactions across a wide range of sites, particularly addressing the limitations on marginal land (Karp *et al.*, 2011), including responses to water availability extremes.

For sandy soils in northern latitudes the success of SRC willow has been attributed to early growth under low evapotranspiration (ET) demand and high precipitation (Larsen *et al.*, 2014). Depending on water availability, ET during the summer months can range from 365 to 495 mm (Persson, 1995), of which ~80 % is transpiration (Linderson *et al.*, 2007). For extensively managed SRC, with a yield gap of 30 %, water use was estimated to be 68 % of potential ET (Horemans *et al.*, 2017). Hydrological modelling has shown that actual ET of a fully grown SRC plantation can be ~16 % greater than the ET of annual crops, which can reduce groundwater recharge by almost 50 % (Hartwich *et al.*, 2016). More evidence is needed with respect to model parameterization to simulate groundwater recharge of SRC regarding canopy development, stomatal resistance (Richter *et al.*, 2015) and rooting depth (Persson, 1995).

Originally, rapid build-up of leaf area and early canopy closure were found to be effective strategies for biomass accumulation (Sennerby-Forsse, 1995); however, this can result in high transpiration (Karp and Shield, 2008). Breeding new, more productive cultivars with lower water use has targeted morphological traits, including improved canopy structure and light interception (Cerasuolo *et al.*, 2013; Larsen *et al.*, 2014; Weih *et al.*, 2014), which are promising traits for enhancing resource use efficiency (Cerasuolo *et al.*, 2016). These approaches have exploited the greater net photosynthetic capacity of narrow-leaved (NL) *Salix viminalis *compared with broad-leaved (BL) species (Cienciala and Lindroth, 1995), as the ability to produce similar, or greater, biomass at lower leaf area is considered an important yield trait of *Salix* species (Bouman and Sylliboy, 2012). Successful selection for productivity therefore lies in optimizing leaf area, light interception and specific photosynthetic capacity (Andralojc *et al.*, 2014).

If water is limiting, water use efficiency (WUE) becomes an important trait for sustainable yield (Karp and Shield, 2008; Serapiglia *et al.*, 2013) and is a key trait for improving productivity of poplar (Liang *et al.*, 2006) and SRC willow (Lindroth *et al.*, 1994; Bonneau, 2004; Linderson *et al.*, 2007). WUE ranges widely (2.4–8.5 g DM kg^−1^ H_2_O; Linderson *et al.*, 2007) and willow genotypes with high intrinsic (leaf-specific) WUE produce increased shoot biomass under water limitation (Weih and Nordh, 2002; Linderson *et al.*, 2007). Whole-plant WUE is proportional to intrinsic WUE but may also reflect drought adaptation and water conservation. As rooting density varies between willow genotypes (Cunniff *et al.*, 2015; Gregory *et al.*, 2018), they vary in terms of carbon allocated to the roots in response to water availability (Linderson *et al.*, 2007). This enables drought-tolerant willows to withstand dry periods but at the expense of some reduction in DM yield (Lindroth *et al.*, 1994; Wikberg and Ogreni, 2007). Eventually, improved canopy and growth traits need to be evaluated in terms of performance by quantifying trade-offs of physiological WUE, in order to select genotypes best adapted to different environments (Karp *et al.*, 2011). Larsen *et al.* (2014) found significant ranking (site > clone) and interaction for eight different SRC willow clones tested across five different sites in Denmark. Lowest-yielding sites and clones were 51 and 36 % below the respective best and were affected by management.

The overall aim of this study was to find the optimal G × E combination for growing SRC willow to resource the bioeconomy with a low water footprint. Specific objectives were: (1) to determine the cultivar with the highest sustainable productivity, including the least variability under varying environmental constraints (soil × meteorology); and (2) to optimize the water footprint, by selecting phenotypes for a high WUE and low water use, depending on their canopy size and leaf typology. Particular focus was given to productivity on marginal soils and areas of low water availability. Our work combines agronomic productivity with the environmental water footprint challenge and evaluates how management can modify these at the regional and sub-regional scales.

## MATERIALS AND METHODS

### Implementation of the LUCASS model into the simulation modelling framework

All the simulations were performed with the LUCASS (light use and carbon assimilation in *Salix* species) model (Cerasuolo *et al.*, 2016), a process-based crop growth model for SRC willow, able to reflect G × E interaction in its parameter space. It was calibrated in two locations in the UK with and without water stress (at Rothamsted Research, south-east England, and at Aberystwyth, Wales, respectively) using carbon partitioning data of a 2-year rotation following the year of establishment. It was validated at Rothamsted Research for two successive 2-year rotations for stem (dry biomass, number and height), leaves [phenology and leaf area index (LAI)] and stool (dry biomass) development.

#### Plant growth parameters

The model was calibrated for four willow (*Salix*) cultivars: two BL (leaf width 20–27 mm), closed-canopy cultivars, ‘Endurance’ (*S. rehderiana* × *S. dasyclados*) and ‘Terra Nova’ [(*S. viminalis* × *triandra*) × *S. miyabeana*]; and two NL (leaf width 14–19 mm), open-canopy cultivars, ‘Resolution’ (multiple parental crosses of *S. viminalis* × *S. schwerinii*) and ‘Tora’ [*S. schwerinii* × (*S. viminalis × viminalis*)]. It was also validated for final harvest after a 3-year rotation at Rothamsted Research and Long Ashton (south-west England) for ‘Endurance’, ‘Resolution’ and ‘Tora’. Key parameters (Table 1) for phenology, canopy development (maximum LAI and shoot architecture) and root extension show clear differences, especially in terms of leaf width and extension rates, which overall results in smaller canopies in ‘Tora’ and ‘Resolution’ (Cerasuolo *et al.*, 2013). Further detail can be seen in Supplementary Data Table S1.

**Table 1.** Genotypic key parameter values in the process-based model LUCASS

Parameter		‘Endurance’	‘Terra Nova’	‘Resolution’	‘Tora’
Leaf width (m)		0.0276	0.0205	0.0194	0.0137
Leaf shape factor (m m^–1^)		0.78	0.75	0.64	0.59
Clumping index		1.5	1.34	1.25	1.41
Linear leaf extension	Coefficient (m d^−1^)	0.000072	0.00007	0.000085	0.000083
	Constant (m)	5	3.5	2.5	2.5
Fraction of assimilates going to above-ground		0.75	0.75	0.8	0.75
Fraction of belowground assimilates going to roots		0.75	0.7	0.75	0.75
Linear stem elongation	Coefficient (m d^−1^)	0.0078	0.0077	0.0098	0.0095
	Constant (m)	11	10.8	10.8	10.8
Linear relationship of diameter to height	Coefficient (m d^−1^)	5.4	5.1	4.8	4.5
	Constant (m)	0.36	0.49	0.25	0.31
Linear relationship of height to stool weight	Coefficient (m d^−1^)	0.015	0.0163	0.0368	0.0239
	Constant (m)	0.0896	0.0758	0.0218	0.0803
Maximum rooting depth (m)		1.2	1.2	1.2	1.2
Root elongation rate (mm d^−1^)		0.0015	0.0017	0.0017	0.0015
CO_2_ potential assimilation rate at light saturation (g CO_2_ m^−2^ s^−1^)		0.001	0.0011	0.00085	0.00103
Stomatal resistance (m s^−1^)		180	100	196	132

#### Soil water module

Soil water balance, transpiration and water uptake were calculated using the Penman–Monteith equation. The soil water balance was based on the ISBA (Interaction Soil Biosphere Atmosphere) approach (Noilhan and Planton, 1989) as described by Richter *et al.* (2006), with two layers: the top layer (0–0.1 m) and the deep layer (0.1 m; maximum genotypic rooting or soil depth). As the soil water content (SWC) of the top horizon fluctuated greatly due to precipitation events, we evaluated the model using the SWC of the second layer. The hydraulic parameters of the layer (mainly water retention curves) were estimated using a pedotransfer function (see below). The water stress coefficient and its impact on plant development were calculated from the relative SWC of the deep layer by using a logistic function (Richter *et al.*, 2001) derived from a function proposed by Sinclair *et al.* (1987).

#### Data for validation of soil water balance simulation

A field trial was established in a randomized complete block design at Rothamsted Research in 2009 for the four SRC willow cultivars described above (Cunniff *et al.*, 2015). Shrubs were coppiced in January of 2010, 2012, 2014 and 2016. Each plot contained 11 double rows, with a spacing of 0.5 m between the stools in the rows, 0.8 m within each double row (between the two stools in a double row) and 1.6 m between adjacent double rows. Within each plot, one double row was used for non-destructive measurements of the shrub canopy (LAI) using the SunScan Canopy Analysis system, type SS1 (Delta-T Devices Ltd, Cambridge, UK). We measured SWC during the growing period with a capacitance probe (Diviner 2000, Sentek Pty Ltd, Australia) using an access tube installed in the area of non-destructive measurement inside the double rows of each plot. The Diviner probe was calibrated at the establishment in 2009 and adjusted in June 2013 against volumetric soil moisture samples, and SWC simulated with LUCASS was validated using Diviner measurements between 2010 and 2014 (Richard *et al.*, 2015).

### Selection of input data for the scenario simulations

#### Meteorological data

Meteorological daily data (minimal and maximal air temperatures, global solar radiation, precipitation, wind speed and relative humidity; Table 2) were provided by the UK Meteorological Office. These were converted from daily to hourly values, applying sinusoidal functions to temperature, daylength and global radiation (Goudriaan and van Laar, 1994). For rainfall disaggregation we assumed the general validity of local evidence for rainfall duration of 6 h. Two scenario periods were selected to simulate two 24-year willow cultivations: a ‘baseline’ period (1965–89) and a ‘recent’ period (1990–2014). The UK was divided into four climatic areas with two weather stations per area (Supplementary Data Fig. S1). The north-west (NW) was defined as a wet, cool area with the stations of Belfast (Northern Ireland) and Carlisle (NW England) used for both scenario periods, the north-east (NE) as a dry, cool area with data from Aberdeen (Scotland) and High Mowthorpe (NE England), the south-east (SE) as a dry, warm area with data from Rothamsted Research (Hertfordshire) and Oxford (Oxfordshire), and the south-west (SW) as a wet, warm area with data from Aberporth (Wales) and Plymouth and North Wyke (Devon) for the baseline and recent scenarios, respectively.

**Table 2.** Averages of daily mean air temperatures, annual precipitation and annual global radiation between 1965 and 1989 (baseline) and between 1990 and 2014 (recent) in four climatic regions in the UK (two weather stations per area). The differences between recent and baseline scenario values are represented in parentheses

	Air temperature (°C)		Precipitation (mm)		Global radiation (MW m^−2^)	
Region	Baseline	Recent	Baseline	Recent	Baseline	Recent
NW	8.69	9.41 (+0.71)	860	875 (+15)	746.6	851.8 (+105.2)
NE	7.76	8.64 (+0.88)	788	781 (-7)	793.9	856.2 (+62.4)
SW	9.86	9.93 (+0.06)	931	973 (+42)	912	1008.2 (+96.3)
SE	9.39	10.34 (+0.95)	676	680 (+4)	909.1	988.5 (+79.4)

The data show a clear separation of the regions and a general warming trend (Table 2). The low increase in temperature for the SW compared with the other regions may be due to discontinuation of data for Plymouth and the use of data from North Wyke instead for the recent scenario. There was no clear temporal change for rainfall, but higher precipitations in western regions. Overall, global solar radiation increased in all regions in the warmer recent scenarios compared with those of the baseline scenarios. According to the UK Meteorological Office, the years 1973–76, 1988, 1991, 1995, 1996, 2005, 2010 and 2011 were defined as drought years for the UK, which in 2011 mainly affected central England (https://www.metoffice.gov.uk/climate/uk/interesting/2012-drought).

#### Soil selection and parameters

Seventeen soil types with different textures, bulk densities and depths were selected to have a variation of soil available water capacity (SAWC) ranging from 51 to 251 mm (Supplementary Data Table S2). Soil parameters for the hydrological model were derived from texture (sand/silt/clay content), organic matter and bulk density available from the fundamental soil property tables provided in the NATMAP data base (Hallett *et al.*, 2017) using pedotransfer functions (Wösten *et al.*, 1999). The SAWC is the water retained between field capacity and wilting point; water contents for field capacity were estimated at −10 kPa for gleysols and −33 kPa for any other soil, and at −1500 kPa for wilting point. The SAWC is the sum of horizon-specific available water capacity accumulated to depth of rock or maximum rooting. Soils were assumed to represent a range of SAWC and were not necessarily specific to the region.

### Outputs of the model

Scenario simulations were run for the four willow cultivars for both climatic periods and all 17 soils and eight sites for 2-year and 3-year rotations, generating yields and hydrological variables for twelve 2-year and eight 3-year coppicing cycles for each period. For each genotype × site climate × soil, we obtained specific annual values for stem dry biomass (harvestable yield) and various other indicators, such as the minimal water stress coefficient, ET and crop yield-related WUE (stem dry biomass production divided by ET; Medrano *et al.*, 2015) during the hydrological year (from the previous October to September of the actual year). Because the first year of each scenario does not cover an entire hydrological year, years 1965 and 1990 were removed from the analysis. Only complete whole years (calendar and hydrological) were used for the analysis, corresponding to the growing seasons of 1966–88 (baseline) and 1991–2013 (recent), and 23 production years for each scenario.

### Statistical analysis

Three-way ANOVA (genotype × region × period) and *post hoc* Tukey’s honest significant difference (HSD) tests (*P* < 0.05) were performed using the statistical software R with the stats package (R Core Team, 2017) on annual yields and WUE after assertion of normality of the data. Distribution of annual yields, WUE and empirical cumulative distribution frequencies were also calculated with the stats package of the software R.

The hydrological model performance was evaluated with the root mean squared error (RMSE) and the Nash–Sutcliffe modelling efficiency index (EF):

$$\begin{array}{l} RMSE=\;\sqrt{\frac{\overset{n}{\underset{i=1}{\sum }}{\left(S_{i}-O_{i}\right)}^{2}}{n}} \end{array}$$

where $S_{i}$ and $O_{i}$ are the simulated and average measured SWC at date i, respectively, and n is the number of measurement dates.

$$\begin{array}{l} EF=1-\;\frac{\overset{n}{\underset{i=1}{\sum }}{\left(S_{i}-O_{i}\right)}^{2}}{\overset{n}{\underset{i=1}{\sum }}{\left(\bar{\;O}-O_{i}\right)}^{2}} \end{array}$$

where $\bar{\;O}$ is the average measured SWC of all measurements.

## RESULTS

### Evaluation of the model against observed soil moisture data at Rothamsted Research

Observations in the 0.1-m maximum rooting depth soil layer during the growing season showed a decrease in SWC (and so an increase in water extraction) with an increase in LAI. The extraction was greater for the BL (‘Endurance’ and ‘Terra Nova’) than for the NL (‘Resolution’ and ‘Tora’) cultivars (Richard *et al.*, 2015). The model accurately simulated both decrease in SWC during the growing seasons for all cultivars and the greater extraction of soil water for BL than NL cultivars during these periods (Supplementary Data Fig. S2). Moreover, the model succeeded in simulating differences between dry and wet years. Apart from ‘Resolution’, the model efficiency was >0.58 and the RMSE varied between 0.027 m^3^ m^−3^ for ‘Tora’ and 0.044 m^3^ m^−3^ for ‘Resolution’ during the total validation period (2010–16, Supplementary Data Table S3).

### Effects of climate on productivity

The results showed regional and temporal differences, which are driven by global radiation, air temperature, water availability and drought controlled by a combination of precipitation and SAWC. Because these regional and temporal patterns were similar between 2- and 3-year growth cycles, only 2-year averages will be discussed except where specified.

#### Regional G × E effects

Under the baseline climate scenario, lower yields were simulated in the (cooler) north than in the south, and in the (drier) east than in the west. Overall, the different phenotypes showed a consistent ranking of productivity (‘Endurance’ > ‘Terra Nova’ > ‘Resolution’ > ‘Tora’), which was found to be significant in the north (Tukey HSD, *P* < 0.05). In the south, only yields for the NL cultivar ‘Tora’ were significantly lower, while the NL cultivar ‘Resolution’ was overall as productive as the highest-yielding cultivar, ‘Endurance’ (Table 3). Under the warmer recent climate scenario, simulated productivity was highest in the disproportionally warmer maritime NW. The ranking of phenotypes remained approximately the same but the difference between the NL and BL cultivars was smaller. In the SE, which is the area most likely to suffer from drought, the NL cultivar ‘Resolution’ was superior, also in terms of reduced yield variation across all sites (soils).

**Table 3.** Average simulated annual yields (stem DM production) and WUE of four willow genotypes in a 2-year growth cycle under baseline and recent climate in four regions in the UK. Lowercase letters indicate significant differences (*P* < 0.05, Tukey HSD test)

	Scenario	Baseline		Recent		Baseline		Recent	
Region	Genotype	Simulated annual yield (t ha^−1^)				Simulated WUE (g DM kg^−1^ H_2_O)			
NW	‘Endurance’	10.2 (±2.2)	abc	12.0 (±2.8)	n	3.7 (±0.5)	abc	3.8 (±0.5)	k
	‘Terra Nova’	9.5 (±1.9)	de	11.2 (±2.2)	hijop	3.5 (±0.4)	def	3.5 (±0.4)	dn
	‘Resolution’	9.3 (±1.6)	e	11.0 (±1.8)	hijkp	3.5 (±0.4)	def	3.6 (±0.3)	ilmn
	‘Tora’	8.1 (±2.1)	f	10.6 (±1.8)	aklm	3.3 (±0.4)	g	3.5 (±0.3)	dmn
NE	‘Endurance’	10.0 (±2.3)	bcd	11.4 (±3.1)	opq	3.6 (±0.6)	bchi	3.8 (±0.6)	k
	‘Terra Nova’	9.3 (±1.8)	e	10.6 (±2.5)	aklm	3.4 (±0.5)	fj	3.5 (±0.5)	dmn
	‘Resolution’	8.8 (±1.7)	g	10.2 (±2.2)	abc	3.3 (±0.4)	gj	3.5 (±0.4)	den
	‘Tora’	8.2 (±1.5)	f	9.9 (±2.0)	cd	3.3 (±0.4)	gj	3.5 (±0.3)	dlmn
SW	‘Endurance’	11.0 (±2.9)	hijkl	11.7 (±3.1)	nq	3.7 (±0.6)	abch	3.7 (±0.6)	abch
	‘Terra Nova’	10.9 (±2.3)	ijkl	11.5 (±2.6)	oq	3.6 (±0.6)	bchi	3.5 (±0.6)	dlmn
	‘Resolution’	10.8 (±2.1)	jklm	11.3 (±2.3)	hiopq	3.7 (±0.4)	abk	3.6 (±0.5)	chilm
	‘Tora’	10.3 (±1.7)	abc	11.2 (±2.0)	hijop	3.7 (±0.4)	ak	3.6 (±0.5)	abchi
SE	‘Endurance’	10.7 (±2.8)	aklm	11.3 (±3.1)	hiopq	3.5 (±0.5)	dlmn	3.6 (±0.6)	chil
	‘Terra Nova’	10.4 (±2.2)	am	11.1 (±2.6)	hijop	3.5 (±0.5)	def	3.6 (±0.5)	hilmn
	‘Resolution’	10.4 (±2.1)	abm	11.4 (±2.5)	hopq	3.6 (±0.4)	hilmn	3.8 (±0.4)	k
	‘Tora’	9.4 (±1.9)	e	10.6 (±2.4)	alm	3.4 (±0.4)	efj	3.6 (±0.5)	chil

#### Climate-change effects on distribution of simulated yield

**Fig. 1.**
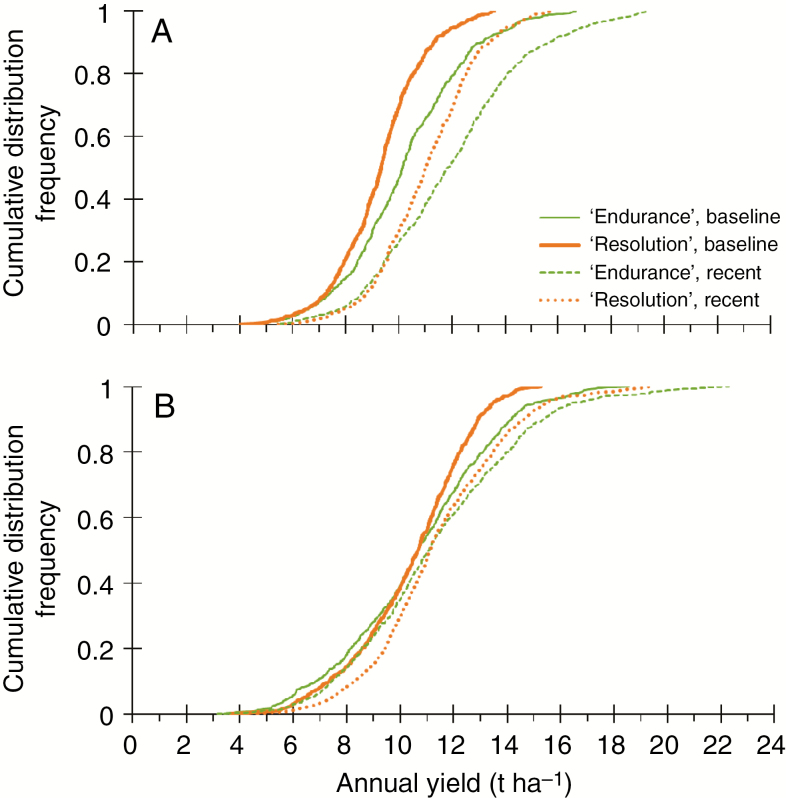
Empirical cumulative distribution frequencies of simulated annual yields for willow cultivars ‘Endurance’ and ‘Resolution’ for a baseline (1966–88) and a recent (1991–2013) scenario in the NW (A) and SE (B) of the UK. Each region includes two sites and 17 soils.

Due to increases in temperature for all regions, but especially for the NW, as rainfall remained similar (Table 2), our scenario simulations resulted in overall greater yields (+0.6 to 1.8 t ha^−1^). The shift to greater yield distributions was more accentuated in the north (Fig. 1A) than in the south (Fig. 1B). Overall, the distribution of simulated yields of ‘Endurance’ was wider than for ‘Resolution’, as expressed in a lower slope. The fraction of yields above the economic threshold of 9 t DM ha^−1^, above which profits are to be expected, increased from 60–70 % under the baseline to ~ 85 % for both phenotypes under the recent scenario in the NW (Fig. 1A). In the drought-prone SE the fraction below the threshold was larger for ‘Endurance’ than for ‘Resolution’. For ‘Tora’, the other NL cultivar, the fraction above the economic yield increased from 0.28 to 0.71 and from 0.32 to 0.82 in the NE and NW, respectively (Supplementary Data Table S4).

### Effect of soil quality (SAWC) on productivity and water use

#### Water availability of arable soils

**Fig. 2.**
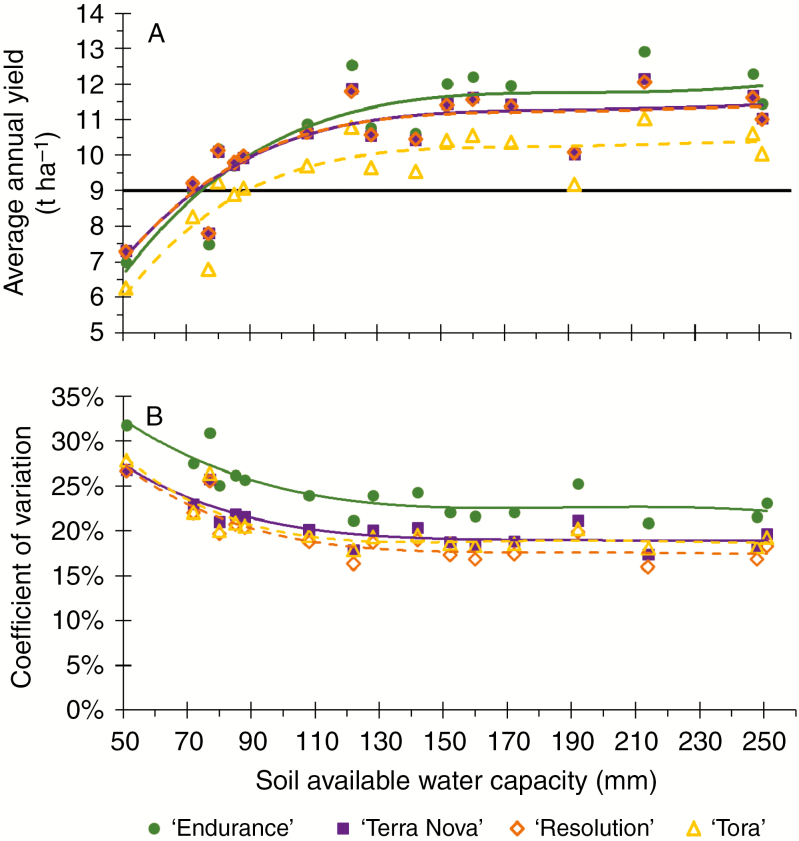
Average simulated annual yields (A) and associated coefficients of variation (B) for willow cultivars ‘Endurance’ (green filled circles), ‘Terra Nova’ (purple filled squares), ‘Resolution’ (orange open diamonds) and ‘Tora’ (yellow open triangles) for 2-year growth cycle management between 1966 and 1988 (started in 1965) for two weather stations in the SE of the UK regarding the SAWC of 17 soils. An economic threshold of 9 t ha^−1^, above which profits are made, is represented by a black line and coloured lines represent polynomial regressions of the genotypic values.

For all four regions, average simulated yields were low in soils with SAWC <100 mm, and the differences between the genotypes ‘Endurance’, ‘Terra Nova’ and ‘Resolution’ were minor, whilst ‘Tora’ failed more clearly in the SE (Fig. 2). In the northern regions the spread between NL and BL cultivars was large under the baseline scenario (data not shown) due to lack of radiation and lower temperatures (Table 2). The economic threshold of 9 t DM ha^−1^ was hardly ever exceeded by the NL phenotype ‘Tora’ in the northern latitudes but was exceeded by all others. This changed under warmer recent climate (Supplementary Data Fig. S3).

Under recent climate scenario simulations, all cultivars are likely to reach or exceed the economic threshold at greater SAWC, although in the cool, dry NE all NL cultivars struggled to grow beyond the threshold. Under a cool climate, with lower radiation, larger canopies (BL) were clearly superior to small (NL) canopies according our model. In the warmer south all cultivars produced within a similar range (Table 3). However, in the SE, where drought stress is more likely than in the other regions, the NL cultivar ‘Resolution’ was found to be only marginally superior to ‘Endurance’, but much less variable when grown on soils with a low SAWC (<100 mm; Fig. 2A). This phenomenon of an overall smaller yield variation among NL compared with BL genotypes cannot be generalized. However, the coefficient of variation (Fig. 2B and Supplementary Data Fig. S3D–F) for simulated yields was found to decrease from values between 23 and 32 % at 51 mm SAWC by 5–10 % with increasing SAWC. Overall, NL were less variable than BL cultivars.

#### Water use and WUE

**Fig. 3.**
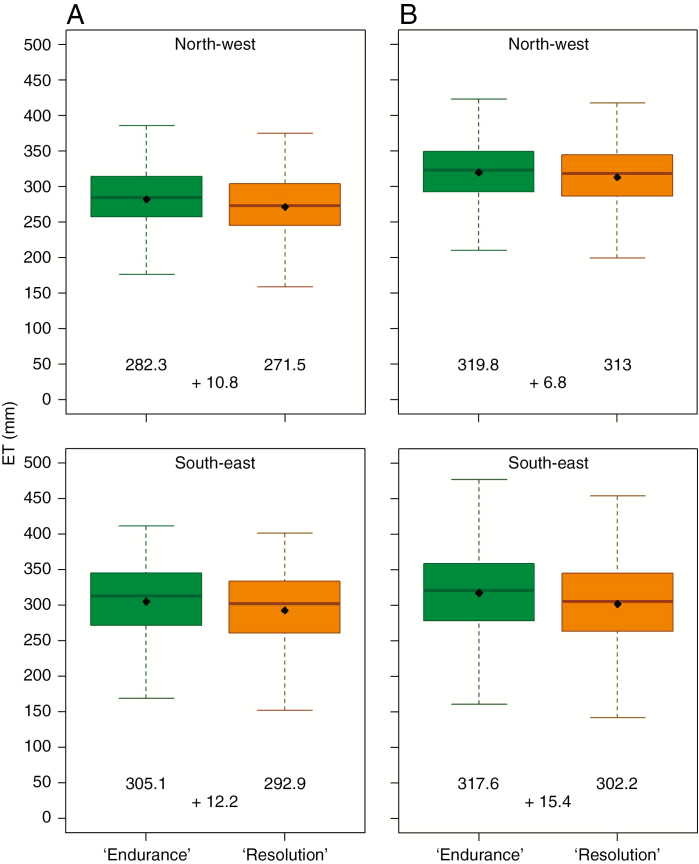
Cumulative simulated ET from October in the previous year to September for SRC willow cultivars ‘Endurance’ (green) and ‘Resolution (orange) and a 2-year growth cycle management in the NW (top) and SE (bottom) of the UK (two weather stations and 17 soils per region) between 1966 and 1988 (baseline, A) and 1991 and 2013 (recent, B). Averages are represented by a black dot; under each boxplot is shown their values and the differences between ‘Endurance’ and ‘Resolution’ means are shown between them. The bottom and the top of the box represent the first (Q1) and third (Q3) quartiles, respectively, and the median is represented by the thick line inside the box. The lower and upper whiskers equal Q1 − 1.5 × (Q3 − Q1) and Q3 + 1.5 × (Q3 − Q1), respectively.

Under baseline weather, modelled water use (ET) ranged under water limitation from 150 to 440 mm in the SW and from 175 to 375 mm in the NW/NE, with some extremely low annual ET rates of <150 mm for the BL cultivar ‘Endurance’ (Fig. 3 and Supplementary Fig. S4). Under identical conditions, simulated ET for ‘Resolution’ with its slightly smaller canopy was ~10 mm lower. Under the warmer recent scenario simulations, ET of ‘Endurance’ was on average ~40 and 20 mm greater than that of ‘Resolution’ in the north and south, respectively. The average increase in simulated ET was the smallest in SE England but with the greatest variation, ranging from ~100 to 475 mm. Differences between canopy phenotypes were found to be the largest in the SE (~15 mm), reflecting the water saving potential of NL phenotype ‘Resolution’.

**Fig. 4.**
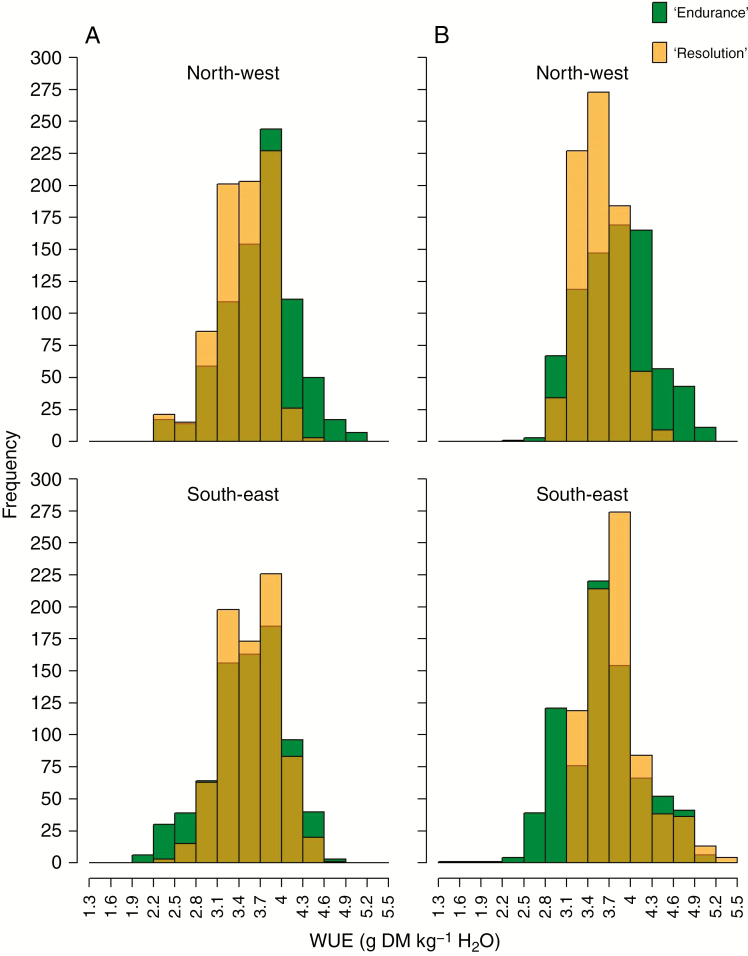
Simulated WUE distribution comparison between ‘Endurance’ and ‘Resolution’ for baseline (A, 1966–88) and recent (B, 1991–2013) scenarios in the NW (top) and SE (bottom) of the UK (two weather stations and 17 soils per region).

Overall, simulated WUE ranged from <2 to >5.2 g DM kg^−1^ H2O (Fig. 4 and Supplementary Data Fig. S5). WUE showed a greater variation for ‘Endurance’ compared with ‘Resolution’, meaning that ‘Resolution’ was superior in terms of water economy (Table 3). In terms of regional distribution, WUE in the north was more frequently found to be greater for ‘Endurance’ than for ‘Resolution’. In contrast, WUE in the south was typically lower for ‘Endurance’ than for ‘Resolution’. In the northern regions, WUE was greater for ‘Endurance’ than for the three other cultivars; in the SW, WUE was greater for NL phenotypes (‘Resolution’ and ‘Tora’) than for BL ‘Endurance’ (baseline only) and ‘Terra Nova’ (both periods); in the SE, WUE was highest for ‘Resolution’ (but the difference in comparison to ‘Endurance’ was significant only in the recent scenario; Tukey HSD, *P* < 0.05).

#### Marginal soils in terms of droughtiness

**Fig. 5.**
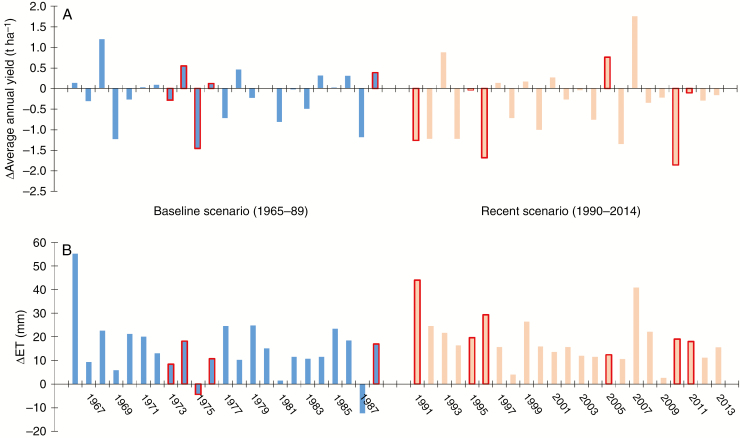
Differences (*Δ*) in annual simulated average yield (A) and ET from October the previous year to September (B) between two willow cultivars (‘Endurance’ minus ‘Resolution’) on marginal soils (SAWC <100 mm) between 1966 and 1988 (started in 1965, in blue) and between 1991 and 2013 (started in 1990, in light red) and a 2-year growth cycle management in the SE of the UK (average of two sites).1 The red borders represent drought years according to the UK Meteorological Office.

As shown above (Fig. 2), the lowest and most variable modelled yields were identified on marginal soils characterized by low SAWC. In these soils, ‘Resolution’ is likely to outperform ‘Endurance’, resulting in greater WUE. The examples displayed in Fig. 5 represent the simulated response of ‘Endurance’ (BL) and ‘Resolution’ (NL), with a clear win–win in terms of productivity and water use in the more drought-prone SE. The modelled average yield (*Y*) difference [*ΔY* = *Y*(BL) – *Y*(NL)] was superior (i.e. *ΔY* < 0) for the NL cultivar ‘Resolution’, in terms of both frequency and magnitude. A significant yield advantage (*ΔY* < −1 t ha^−1^) was likely to be twice as frequent and doubled on average (−0.37 versus −0.15 t ha^−1^) under the warmer recent weather scenario. In addition, ‘Resolution’ saved water, as its average simulated ET was 24 mm lower in drought-prone years.

Except for two out of 46 years, ‘Resolution’ could have given an annual water saving of between 5 and 55 L m^−2^ when compared with ‘Endurance’. On average, growing ‘Resolution’ on marginal soils could virtually save water annually at 16.5 L m^−2^, which would correspond to 1 650 000 m^3^ of water, assuming willow plantations of 10 000 ha.

#### Effect of coppice management and length of growth cycle

**Fig. 6.**
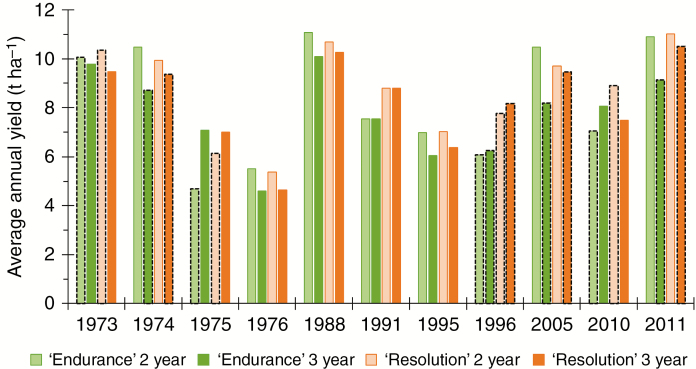
Average simulated annual yields for willow cultivars ‘Endurance’ (green) and ‘Resolution’ (orange) and 2-year (light colour) and 3-year (dark colour) growth cycle management on marginal soils (SAWC <100 mm) at two weather stations in the SE of the UK for drought years as defined by the UK Meteorological Office. Black dashed borders represent the first year of a growth cycle.

We addressed the question of whether, beyond selection of cultivar × location, other management options might reduce the risk of low yields on marginal soils. Figure 6 focuses on the effect of SRC plantations managed in 2- or 3-year rotations for droughty years in the SE. Usually, for the same year lower yields were simulated for a cultivar when it was a first year of (re)growth compared with a second or third year in the other management system. Greater management effects were found for ‘Endurance’ as drought during the year after coppicing impacted more strongly on BL canopy development (‘Endurance’) than on the NL type (‘Resolution’). In conclusion, SRC with a small NL canopy phenotype (e.g. ‘Resolution’) is the superior choice on marginal soils, particularly under a dry climate, for achieving sustainable yield and lower water use.

## DISCUSSION

The climate change scenario analysis for SRC willow plantations presented here is based on the combination of a validated G × E optimization model (Cerasuolo *et al.*, 2016) with real climate data showing a recent temperature increase of up to 1 °C. The simulation scenario results highlight three key phenomena and criteria to consider: (1) phenotypic canopy size and architecture for light- or water-limited environments; (2) water use and WUE to mitigate water shortage (saving water) or to increase productivity under water shortage; and (3) the economics of bioeconomy and climate mitigation.

### Phenotyping for canopy optimum (NL versus BL) under climate change

In our model, photosynthesis, growth and transpiration are tightly linked through multiple parameters representing traits that modify WUE across different scales from leaf to plant to field scale (Medrano *et al.*, 2015). The intrinsic WUE (ratio between net photosynthesis and transpiration), controlled by stomatal conductance and abscisic acid (Gago *et al.*, 2014), is simplified in our model using a simple water-stress response function. The simulated reduction factor (*k*_*ws*_) regulates photosynthesis and growth in the model, which enables the simulation of regional-scale climatic scenarios (Supplementary Data Fig. S6). The simulation results show that severe growth reduction (*k*_*ws*_ < 0.5) was more likely for ‘Endurance’ (BL) than for ‘Resolution’ (NL), reflecting greater water use and stress for the cultivar with BL traits forming a large canopy. For light interception, Cerasuolo *et al.* (2013) described the importance of these leaf traits, which translated into observed and simulated high productivity of the NL type under drought in the experiments during 2010–11 (Cunniff *et al.*, 2015; Cerasuolo *et al.*, 2016). Moreover, the sensitivity analysis performed by Cerasuolo *et al.* (2016) on all the parameters of the LUCASS model we used showed that the modelled differences in yield performance were mainly due to leaf characteristics (such as leaf elongation rate) and parameters related to light interception, and not to other critical morphological or physiological parameters, such as root–shoot ratio. On the contrary, Bonosi *et al.* (2010) did not observe a direct relationship between growth traits and leaf traits among 15 *Salix* genotypes. However, these authors found a significant genotype × water treatment (drought) interaction for plant biomass, height and leaf angle, which confirmed a potential relevance for breeding. Various greenhouse experiments confirmed variable responses to different forms of water stress, expressing large genetic variability for drought tolerance and WUE, found earlier within the *Salix* genus (Ronnberg-Wastljung *et al.*, 2005; Smart *et al.*, 2005; Weih *et al.*, 2006; Kuzovkina and Volk, 2009). Our simulation results confirm that knowing the drought response of biomass willows is crucial for breeding, especially, when they are to be introduced in dry regions susceptible to drought and likely to become even drier in future, e.g. SE England. On the other hand, our simulations showed clearly that, under light limitation, the large canopy of the BL phenotype ‘Endurance’ is likely to be superior in terms of productivity (Fig. 1) and WUE (Fig. 4).

Overall, crop yield and WUE may vary between species and cultivars. Tallis *et al.* (2013) simulated WUE for SRC poplar and willow cultivars in the UK, finding that the poplar ‘Trichobel’ was more water-use-efficient than the willow cultivar ‘Joruun’. However, newer cultivars like ‘Tora’ have a WUE two to three times greater than ‘Joruun’ (Linderson *et al.*, 2007). Therefore, we can conclude that breeding has already successfully improved the WUE of SRC cultivars. Even more recent cultivars, like ‘Resolution’, displayed both high productivity and WUE irrespective of site (Toillon *et al.*, 2013); however, the plasticity in the observed variation for most traits was found to be wide and these results need to be verified in long-term rotations. WUE also seemed to be under the control of stomatal conductance. It is possible that bigger shrubs with larger leaf areas were more subject to periodical droughts during the whole growing season, which might, in turn, cause stomata to close periodically to sustain the water balance (Wikberg and Ogreni, 2007).

### Water stress evasion and savings

Our scenario simulation analysis for a 1 °C temperature increase has shown that, overall, NL-canopy cultivars consume less water than the highly productive BL ones (Fig. 3). In the drought-prone area of SE England on soils of low SAWC the advantage was even bigger (Fig. 5). Climate change and soil degradation could exacerbate mitigation needs due to shifting precipitation patterns, in areas where perennial bioenergy crops should be advantageous. Increasing temperatures may extend the growing season and areas for cultivation (Tuck *et al.*, 2006; DOE, 2013) and increase productivity in the north (Table 3).

Water demand based on WUE and production (Lindroth *et al.*, 1994) at a production rate of 10–12 t DM ha^−1^ corresponds to a transpiration of 286–365 mm of water. This is less than what was modelled under Polish conditions (500–623 mm), which exceeded grass and wheat by 58 and 93 mm, respectively (Borek *et al.*, 2010). However, Borek *et al.* (2010) found that the water footprint per unit biomass or energy of SRC willow was smaller than that for arable crops. Past water use estimates for SRC willow assumed that its root system reaches deeper than the root systems of arable crops (Finch *et al.*, 2004); however, SRC willow root depth can vary considerably (Persson, 1995). As we show in our simulation scenarios (Effect of soil quality [SAWC] on productivity and water use), ET can vary widely, and the modelled difference between NL and BL cultivars of 5–55 mm (Fig. 5) compares well to the 20-mm difference observed in transpiration found for the NL/BL cultivars ‘Tora’ and ‘Loden’ (Linderson *et al.*, 2007). It also compares well to the groundwater recharge reduction (2–24 mm) from the conversion from arable crops to SRC willow plantations simulated across a wide range of groundwater recharge regimes (125–430 mm) by Hartwich *et al.* (2016). In the latter study, it was concluded that hydrological changes of <10 mm can be considered to be of non-substantial impact. However, annual water savings (average of 16 mm year^−1^) due to changes in canopy size, together with greater specific impact when allocated to droughty soils and years (24 mm year^−1^), are likely to be of substantial benefit. The potential of reducing the water footprint became clearer when compared with the range of 23–68 mm in groundwater recharge in dry years (Richter *et al.*, 2015). Overall, the simulated average savings of 16.5 mm for NL cultivar ‘Resolution’ compared with the BL cultivar ‘Endurance’ and average savings of 24 mm (range 12–44 mm) in droughty years are a substantial saving, which is likely to make a increasing difference in a warming climate.

### SRC willow for sustainable land management

Decisions on the use of marginal land to grow dedicated bioenergy crops are complex. SRC willow yields on marginal soils could be as low as 3 t ha^−1^ or <20 % of yields on higher-quality soil (Krzyżaniak *et al.*, 2015), which is well approximated in our simulated distributions (Fig. 1). The advantage of growing NL-canopy phenotypes became particularly visible when production was simulated on hydrologically marginal soils (SAWC <100 mm), and so SRC phenotypes with a small NL canopy (e.g. ‘Resolution’) are the best choice on marginal soils and under dry climates for sustainable yield and lower water use. The difference between 2- and 3-year rotation cycles (Fig. 6) implies a probability that drought hits more frequently during the first year of rotation in a 2-year rotation and so have more chance of impact on the whole rotation, as shown by our results. In particular, early drought has a negative impact on canopy development and usually results in lowers yields, which, in turn, will have greater impact for a cultivar with a larger canopy. Our results suggest choosing a 3-year rotation management to decrease drought risk and its impact on canopy regrowth after coppicing to improve the performance of small canopy phenotypes on marginal soils under dry conditions.

In conclusion, our scenario simulation analysis for mitigating the impact of climate change on SRC plantations in the UK assessed two different commercially grown canopy phenotypes (BL and NL cultivars) in terms of production and water use. These high-yielding cultivars can be regionally selected for carbon capture and water use as follows.

(1) For (up to 1 °C) warmer and more drought-prone climates and regions (e.g. SE England) NL phenotypes with high photosynthetic capacity (e.g. ‘Resolution’) are advantageous due to high yield with low variation and reduced water consumption (delaying drought stress).
(2) For areas with more available water and lower evaporative demands, not affected by occasional drought (e.g. NW UK) BL cultivars with greater light interception and light use efficiency, such as ‘Endurance’, are superior to NL cultivars, such as ‘Resolution’, in terms of yield.
(3) On marginal land with high drought frequency and severity the use of NL cultivars like ‘Resolution’ is superior in terms of yield (up to 2 t ha^−1^) and water use. High-productivity NL cultivars can save >20 mm of water in droughty years, which is a considerable resource with expanding plantations and changing climate.

## SUPPLEMENTARY DATA

Supplementary data are available online at https://academic.oup.com/aob and consist of the following. Table S1: leaf and canopy characteristics of the four SRC willow cultivars used in this study. Table S2: characteristics of the 17 soil types used in the scenario simulations with the process-based model LUCASS for each site. Table S3: hydrological model evaluation for four willow cultivars at Rothamsted Research between 2010 and 2016. Table S4: percentage of years (out of 23 years) to have an annual yield higher than an economical threshold of 9 t ha^−1^ for four regions in the UK and four willow cultivars estimated for two sites per region and 17 soils per sites for the baseline and recent scenarios. Figure S1: weather station locations and partitioning of the climatic areas in the UK. Figure S2: Simulated and observed SWC between 0.1 m and rooting depth, and simulated ET for the willow cultivars ‘Endurance’ and ‘Tora’ at Rothamsted Research in 2011. Figure S3: average modelled annual yields and associated coefficients of variation of four willow cultivars for a 2-year growth cycle management between 1966 and 1988 (started in 1965) for two weather stations in the NW, NE and SW of the UK regarding the soil water capacities of 17 soils. Figure S4: cumulative simulated ET of SRC willow cultivars ‘Endurance’ and ‘Resolution’ and a 2-year growth cycle management in the NE and SW of the UK (two weather stations and 17 soils per region) between 1966 and 1988 and between 1991 and 2013. Figure S5: modelled WUE distribution comparison between ‘Endurance’ and ‘Resolution’ for baseline and recent scenarios in the NE and SW of the UK (two weather stations and 17 soils per region). Figure S6: Frequencies of the annual minimum water-stress coefficient values simulated in the NW and SE for the baseline and recent scenarios for the willow cultivars ‘Endurance’ and ‘Resolution’.

mcz006_suppl_Supplementary_Material
