# Supplementary material for: Optimizing the bioenergy water footprint by selecting SRC willow canopy phenotypes: regional scenario simulations
Source: Ann Bot. 2019 Feb 13;124(4):531–42. doi: 10.1093/aob/mcz006 (PMC6821185; doi:10.1093/aob/mcz006)

# Supplementary data Tables

**Table S1:** Leaf and canopy characteristics of four SRC willow cultivars used in this study. The leaf length to width ratio was measured by planimeter imagery from 10 leaves per cultivar collected from leaf 16 – 25 counting down from first fully emerged leaf at the tip of a strong leading stem.

| **Cultivar** | **Leaf length to width ratio*** | | Ploidy | Parents | Architecture |
| --- | --- | --- | --- | --- | --- |
|  | LA | Roth |  |  |  |
| ‘Endurance’ | 4.22 | 4.92 | Pentaploid | *Salix rehderiana* x  *S. dasyclados* | Multiple stems, broad leaf |
| ‘Terra Nova’ | 5.69 | 5.32 | Triploid | (*S. triandra x viminalis*) x *S. miyabeana* | Multiple stems, broad leaf |
| ‘Resolution’ | 7.93 | 8.40 | Diploid | (*S. viminalis* x Bjorn) x Quest (*S. viminales* x Bjorn) | Fewer stems, narrower leaf |
| ‘Tora’ | 9.93 | 9.59 | Diploid | *S. schwerinii* x *S. viminalis* | Fewer stems, narrower leaf |

*At both sites the difference between leaf length to width ratios was statistically significant (one way ANOVA, F pr. <0.001) with a S.E.D. of 0.418 and 0.410 at Long Ashton (LA) and Rothamsted Research (Roth) respectively.

**Bjorn is a sibling of Tora, i.e. same pedigree.

**Table S2:** Characteristics of the 17 soil types used in the scenario simulations with the process-based model LUCASS for each site.

| Soil name | Depth [m] | SAWC* [mm] | Sand [%] | Silt [%] | Clay [%] | Organic carbon [%] | Bulk density [g cm^-3^] |
| --- | --- | --- | --- | --- | --- | --- | --- |
| Arrowan | 135 | 214 | 14.1 | 68.4 | 17.5 | 1.39 | 1.38 |
| Blackwood | 150 | 128 | 83.6 | 11.7 | 4.6 | 0.80 | 1.42 |
| Blewbury | 55 | 77 | 17.5 | 42.2 | 40.4 | 2.65 | 1.15 |
| Bromsgrove | 130 | 152 | 67.0 | 22.7 | 10.4 | 0.62 | 1.39 |
| Bromyard | 150 | 251 | 9.8 | 70.6 | 19.6 | 0.54 | 1.39 |
| Buriton | 110 | 160 | 19.5 | 59.1 | 21.4 | 0.90 | 1.44 |
| Cranwell | 60 | 80 | 66.1 | 20.7 | 13.3 | 2.19 | 1.24 |
| Elmton | 35 | 51 | 34.7 | 38.7 | 26.6 | 4.81 | 1.01 |
| Escrick | 150 | 192 | 53.9 | 31.6 | 14.5 | 0.72 | 1.42 |
| Frilsham | 60 | 85 | 36.0 | 39.3 | 24.7 | 1.77 | 1.27 |
| Fyfield | 125 | 142 | 67.2 | 19.8 | 12.9 | 0.78 | 1.43 |
| Hendred | 130 | 172 | 8.1 | 51.9 | 40.0 | 0.63 | 1.42 |
| Newport | 150 | 122 | 86.7 | 8.8 | 4.5 | 0.75 | 1.37 |
| Rockcliffe | 150 | 248 | 22.8 | 62.5 | 14.7 | 0.70 | 1.37 |
| Sacrewell | 150 | 108 | 84.1 | 10.5 | 5.4 | 0.66 | 1.39 |
| Soham | 65 | 88 | 50.8 | 27.9 | 21.3 | 1.72 | 1.29 |
| Ston Easton | 50 | 72 | 16.0 | 52.8 | 31.2 | 2.22 | 1.22 |
| *Soil available water capacity | | | | | | | |

**Table S3**. Hydrological model (soil water content, m^3^ m^−3^) evaluation for four willow cultivars at Rothamsted Research between 2010 and 2016: n represents the number of measurements, RMSE the root mean squared error and EF the model efficiency.

|  | Endurance | Terra Nova | Resolution | Tora |
| --- | --- | --- | --- | --- |
| n | 98 | 98 | 98 | 98 |
| RMSE [m^3^ m^−3]^ | 0.034 | 0.031 | 0.044 | 0.027 |
| EF | 0.628 | 0.582 | 0.134 | 0.662 |

**Table S4**. Percentage to have an annual yield higher than an economical threshold of 9 t/ha for four regions in the UK and four willow cultivars estimated for two sites per region and 17 soils per sites for a Baseline (from 1966 to 1988, start in 1965) and Recent (from 1991 to 2013, start in 1990).

|  |  | Percentage of years above 9t ha^-1^ | | | | | |
| --- | --- | --- | --- | --- | --- | --- | --- |
|  |  | 2year-rotation | | | 3year-rotation | | |
| Region | Genotype | Baseline | Recent | ΔR-B | Baseline | Recent | ΔR-B |
| North-West | ‘Endurance’ | 70% | 86% | 16% | 69% | 86% | 17% |
|  | ‘Terra Nova’ | 62% | 84% | 22% | 65% | 90% | 25% |
|  | ‘Resolution’ | 59% | 88% | 28% | 58% | 85% | 27% |
|  | ‘Tora’ | 32% | 82% | 50% | 33% | 82% | 49% |
| North-East | ‘Endurance’ | 67% | 79% | 12% | 71% | 79% | 8% |
|  | ‘Terra Nova’ | 58% | 74% | 16% | 65% | 79% | 13% |
|  | ‘Resolution’ | 49% | 73% | 24% | 50% | 70% | 21% |
|  | ‘Tora’ | 28% | 71% | 43% | 35% | 69% | 35% |
| South-West | ‘Endurance’ | 75% | 82% | 6% | 79% | 81% | 2% |
|  | ‘Terra Nova’ | 79% | 84% | 5% | 84% | 88% | 4% |
|  | ‘Resolution’ | 81% | 85% | 4% | 81% | 86% | 5% |
|  | ‘Tora’ | 79% | 86% | 7% | 78% | 85% | 8% |
| South-East | ‘Endurance’ | 72% | 76% | 4% | 72% | 75% | 2% |
|  | ‘Terra Nova’ | 73% | 78% | 5% | 73% | 76% | 2% |
|  | ‘Resolution’ | 75% | 85% | 10% | 78% | 81% | 3% |
|  | ‘Tora’ | 62% | 77% | 14% | 60% | 72% | 12% |

# Supplementary Data Figures

**
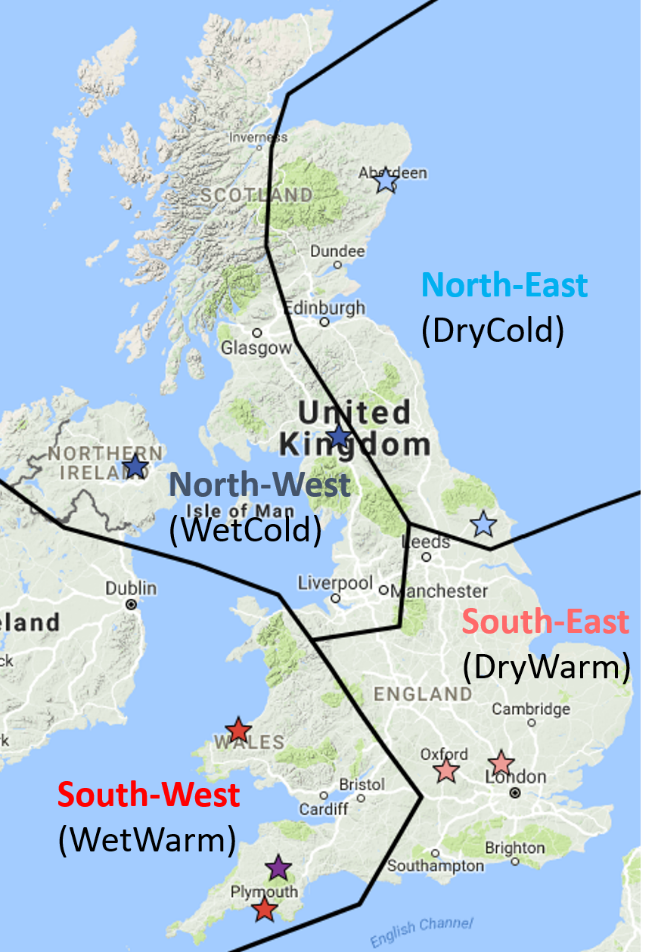
Fig. S1.** Weather station locations and partitioning of the climatic areas in the UK.

**Fig. S2.** Simulated (lines) and observed (symbols) soil water content (m^3^ m^−3^) between 0.1m and rooting depth (A), and simulated ET [mm] (B) for the willow cultivars ‘Endurance’ (green, plain line and circle) and ‘Tora’ (yellow dashed line and open triangle) at Rothamsted Research in 2011.


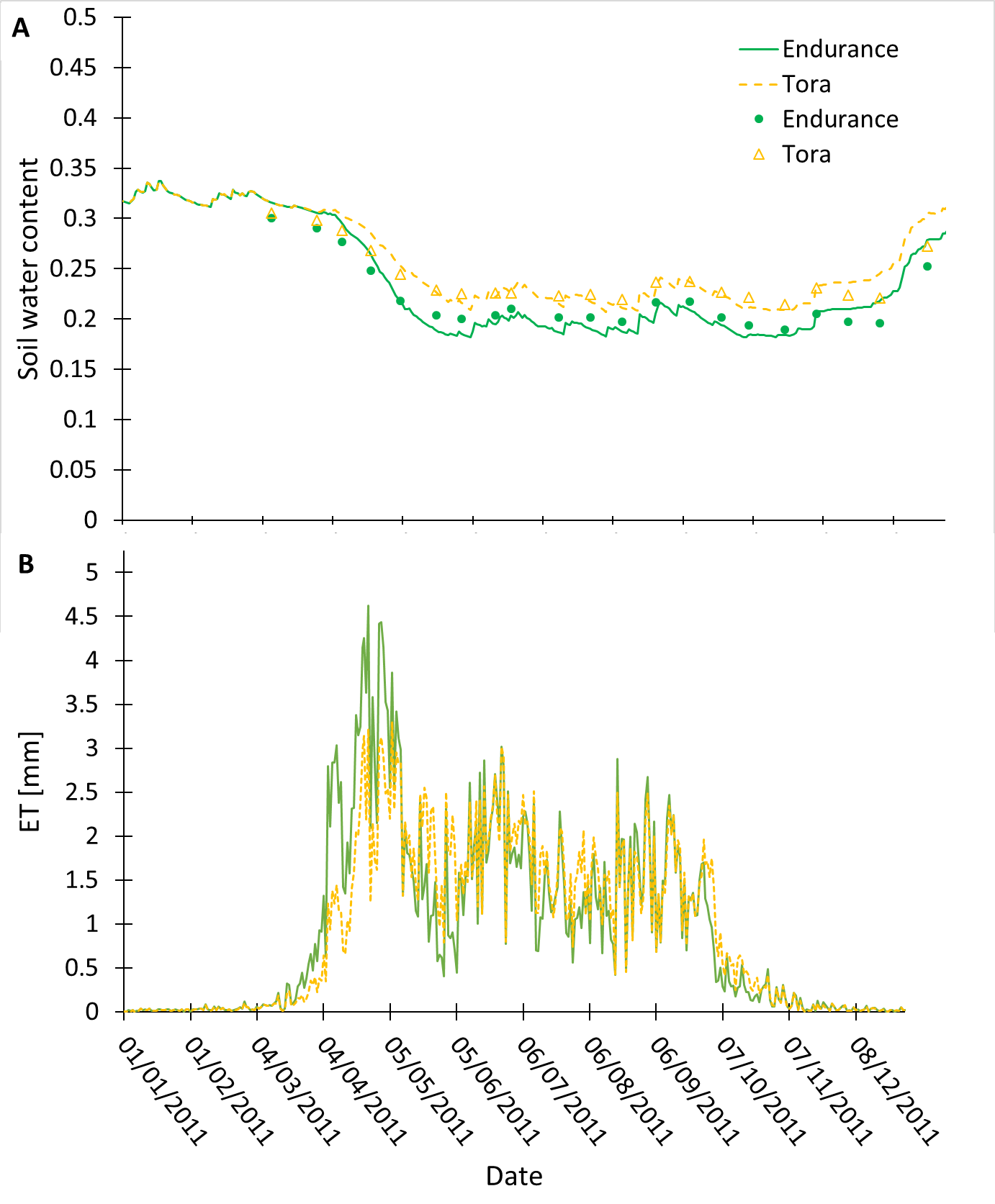


**Fig. S3**. Average modelled annual yields [t ha^-1^] (A-C) and associated coefficients of variations (D-F) of four willow cultivars (‘Endurance’, green filled circle, ‘Terra Nova’, purple filled square, ‘Resolution’, orange open diamond and ‘Tora’, yellow open triangles) for a two-year growth cycle management between 1966 and 1988 (start in 1965) for two weather stations for two weather stations in the North-West (A, D), North-East (B, E) and South-West (C, F) of UK regarding the soil water capacities of 17 soils. An economic threshold of 9 t ha^-1^ above which profits are made is represented by a black line and coloured lines represent polynomial regressions of the genotypic values.

**Fig. S4**. Cumulative simulated evapotranspiration (ET, [mm], from previous year October to September) of two SRC willow cultivars (‘Endurance’ in green and ‘Resolution’ in orange) and a two-year growth cycle management in the North-East (top) and South-West (bottom)of UK (two weather stations and 17 soils per region) between 1966 and 1988 (Baseline, A) and 1991 and 2013 (Recent, B). Averages as represented by a black dot with their values under each boxplot and the difference between ‘Resolution’ and ‘Endurance’ means between them.

**
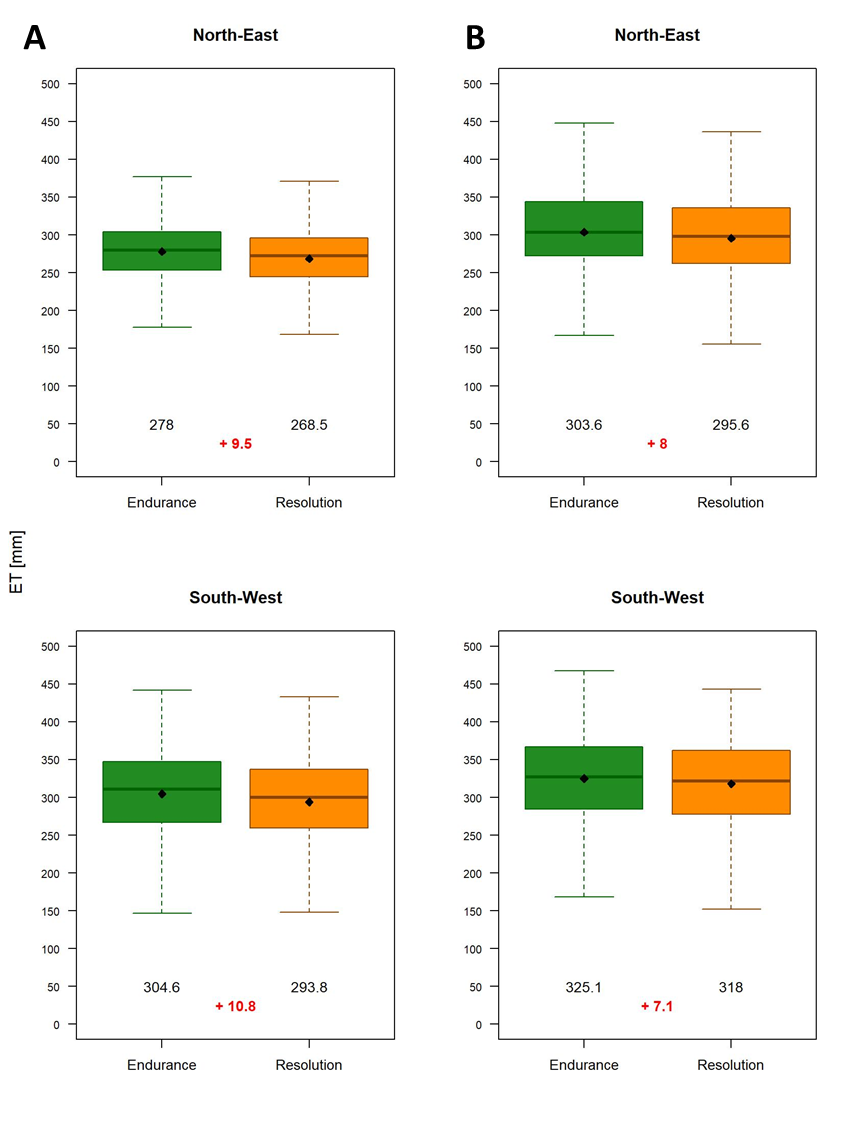
**

**Fig. S5**. Modelled WUE [g DM kg^-1^ H_2_O] distribution comparison between ‘Endurance’ (green) and ‘Resolution’ (orange) for “Baseline” (A, 1966 to 1988) and “Recent” (B, 1991 to 2013) scenarios in the North-East (top) and South-West (bottom) of UK (two weather stations and 17 soils per region)

**
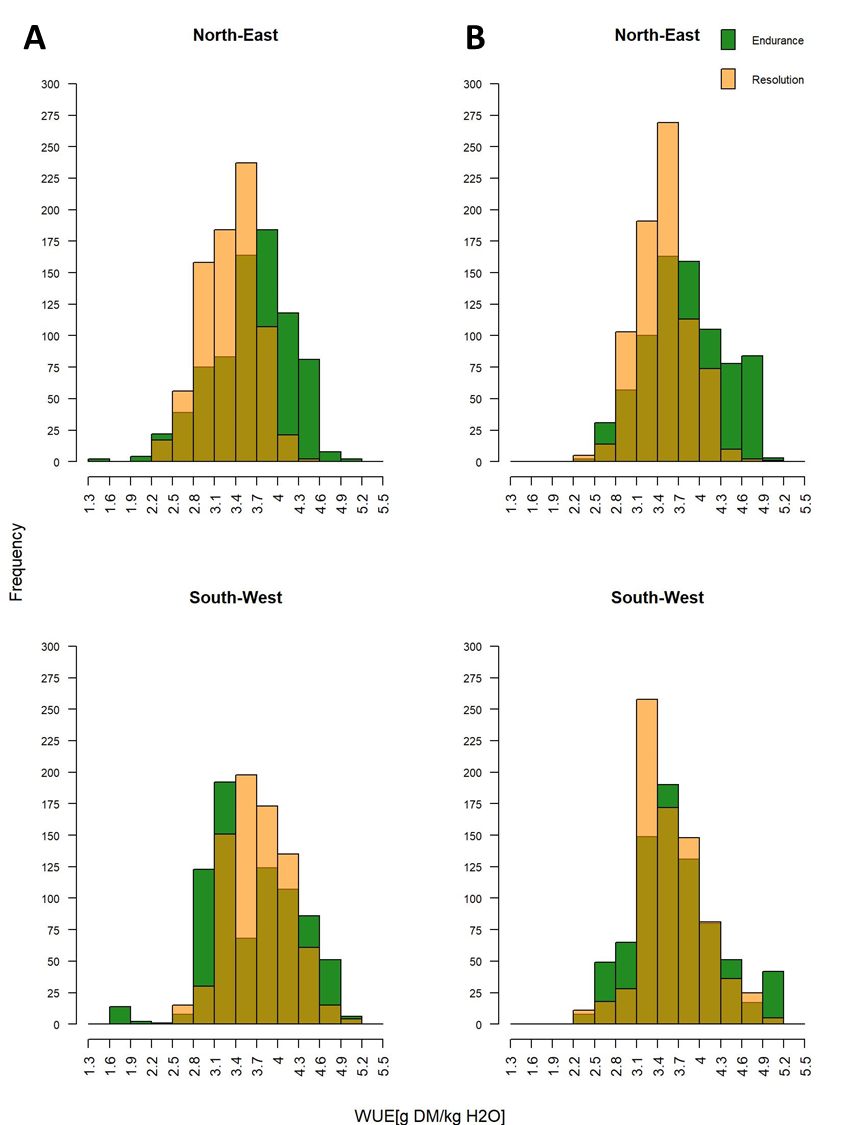
**

**Fig. S6**. Frequencies of the annual minimum water stress coefficient values simulated in the North-West (dark blue) and South-East (light red) for the “Baseline” (A, 1966 to 1988) and “Recent” (B, 1991 to 2013) scenarios for the willow cultivars ‘Endurance’ and ‘Resolution’. The smaller the value is, the higher the water stress is.


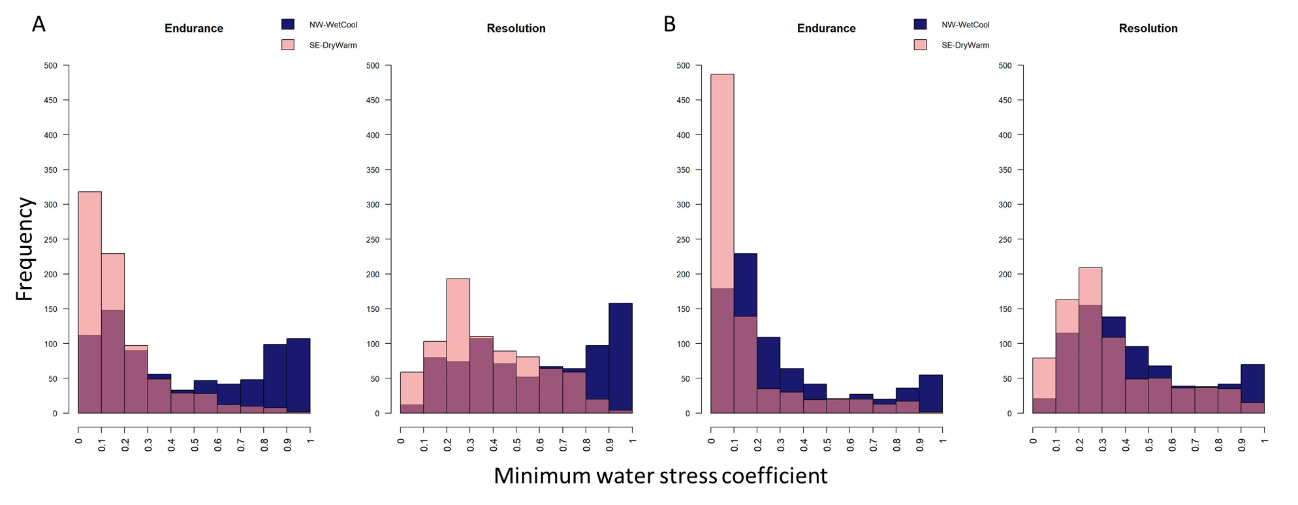

Supplement: mcz006_suppl_Supplementary_Material [file mcz006_suppl_supplementary_material.docx]
